# A Facial Feature and Lip Movement Enhanced Audio-Visual Speech Separation Model

**DOI:** 10.3390/s23218770

**Published:** 2023-10-27

**Authors:** Guizhu Li, Min Fu, Mengnan Sun, Xuefeng Liu, Bing Zheng

**Affiliations:** 1 College of Electronic Engineering, Ocean University of China, Qingdao 266100, China; 2 Sanya Oceanography Institution, Ocean University of China, Sanya 572024, China; 3 College of Automation and Electronic Engineering, Qingdao University of Science and Technology, Qingdao 266061, China

**Keywords:** speech separation, audio-visual, attention mechanism, U-Net

## Abstract

The cocktail party problem can be more effectively addressed by leveraging the speaker’s visual and audio information. This paper proposes a method to improve the audio’s separation using two visual cues: facial features and lip movement. Firstly, residual connections are introduced in the audio separation module to extract detailed features. Secondly, considering the video stream contains information other than the face, which has a minimal correlation with the audio, an attention mechanism is employed in the face module to focus on crucial information. Then, the loss function considers the audio-visual similarity to take advantage of the relationship between audio and visual completely. Experimental results on the public VoxCeleb2 dataset show that the proposed model significantly enhanced SDR, PSEQ, and STOI, especially 4 dB improvements in SDR.

## 1. Introduction

In an environment with multiple sound sources, humans can focus on the target voices and ignore the uninteresting speech or noise depending on the sensitive auditory system, which Cherry defined as the “cocktail party effect” [1]. Speech separation is one of the fundamental tasks for the cocktail party effect, which refers to extracting individual voice signals from a mixed signal of overlapping voices. Speech separation plays a crucial role in numerous applications with the intelligent technology’s rapid growth, such as applying to hearing aids for hearing impairment, operating smart home and mobile phones, speech recognition, and so on. Thus, computational auditory scene analysis (CASA) [2,3], non-negative matrix factorization (NMF) [4], and hidden Markov models (HMM) [5] based on prior knowledge or specific microphone configurations are applied.

Deep learning has been successfully applied to speech separation for decades, substantially advancing the adaptation for wild scenarios by leveraging extensive training data and extracting nonlinear features. The audio-only speech separation (AOSS) methods primarily use audio information to estimate clean speech. In a multiple-speaker environment, assigning sound sources, known as label substitution issues, becomes challenging. Hershey et al. proposed a method called deep clustering (DPCL), in which trained speech embeddings with permutation-free or permutation-invariant loss functions could cluster and separate different sources [6]. Later, some DPCL-based models such as deep attractor network (DANet) [7], ADANet [8], and online deep attractor network (ODANet) [9] are proposed. Because deep learning neural networks (DNN) [10] can obtain more features by multiple hidden layers, recurrent neural networks (RNN) [11], long short-term memory (LSTM) [12] networks, and end-to-end U-Net networks [13] are combined or improved in audio separation successively, such as LSTM RNN association [14], time-domain audio separation network (TasNet) [15], Conv-Tasnet [16], and enhanced Conv-Tasnet [17].

In addition, the ability of humans to focus on specific audio in a cocktail party scene relies not only on the audio but also on the visual information, i.e., the speaker’s gender, age, the motion of the lips, and the speaker’s orientation [18], which has been demonstrated in psychological and biological studies [19,20]. Inspired by this mechanism, some researchers apply neural networks with visual fusion to solve various speech-related problems. The speech separation task that combines visual information is known as audio-visual speech separation (AVSS). On the one hand, looking at a speaker’s face in conversation is beneficial for focusing on the speech [21]. In 2018, Google proposed a speaker-independent audio-visual model for speech separation [22], which uses CNN and LSTM to extract audio and facial features and notably advances the separation performance compared to the audio-only approach. Liu et al. proposed a two-stage feature fusion strategy that can make fuller use of the audio and visual features by fusing the information of high and low-frequency audio features as well as the features of audio and visual [23]. On the other hand, lip motion is related to the content of the voice. Consequently, Wu et al. proposed a lip embedding extractor pre-trained to extract information from the video stream [24], and Lu et al. proposed a model that learned the correspondence between speech and speech fluctuations [25]. Ito et al. mainly conditioned on lip motion and aimed to extract speaker embedding [26]. They proposed an audio-visual speech enhancement (AVSE) model that leveraged a detection and an identification module to retrieve reliable speaker embeddings. However, because these deep learning frameworks are just conditioned on the target speaker’s facial information or lip movements to capture their speech from multiple other speech signals, some features influencing the separation effect have not been extracted sufficiently. Therefore, the audio-visual separation model should consider both static face and dynamic lip features. Deng and Wei proposed a video-guide model that separates audio with the aid of two visual cues and a two-stage strategy that used the speech of the first stage as the second stage’s audio input, which accomplished great separation results [27]. However, the visual information except the face could be redundant, which means that what needs to be focused on is only some essential visual features beneficial for separation.

There exists a strong correlation between visual and audio. Thus, considering this cross-modal link when designing the loss function, we can use the visual feature more comprehensively. Correspondingly, Makishima et al., proposed a cross-modal correspondence loss instead of the mean squared error (MSE) loss to reflect speech and voice characteristics [28]. Li and Qian additionally learned visual representations depending on the attention weight matrix [29]. Lu et al., consider the similarities between audio and video, which aims to learn a shared embedding space to assist speech separation [25], and experimental results show that the separation quality is improved over audio-only methods and verifies the effectiveness of loss. However, how to effectively extract audio and video features and leverage their links is still worth exploring [29].

This paper proposes an audio-visual speech separation architecture that combines visual information and consists of lip, face, and audio separation modules. For audio, residual connections are introduced to distinguish small details and to learn more information, which has been illustrated to be effective in image segmentation [30]. For video, the face module adds the attention mechanism to ensure the most critical face information is taken seriously enough and ignores secondary information. The optimal objective considers the correspondence of audio and video to achieve separation.

## 2. Materials

Assuming that there are two speakers, A and B, in a video, the corresponding time domain discrete forms of their speech signals are represented as $X_{A}$ and $X_{B}$, and then the two signals are mixed as $X_{mix}$:

(1) $$X_{mix}=X_{A}+X_{B}$$

The goal of the speech separation task is to estimate individual $X_{A}$ and $X_{B}$ from the overlapping signals.

### 2.1. Convolutional Neural Network

CNN is a classical deep learning model that uses multiple convolution and pooling operations to extract complex features from the input images while reducing spatial dimensions. A rectified linear unit (ReLU) is introduced to learn complex mappings between input images and output predictions. CNN has been widely applied in many fields because of its ability to learn hierarchical representations directly from raw data without relying on handcrafted features.

#### 2.1.1. U-Net

U-Net is a variant of fully convolutional networks (FCN), and it has the ability of feature extraction for information fused by splicing corresponding pixels [31]. This framework is composed of an encoder and a decoder to capture both the global context and local details effectively, and its structure is shallow and superficial as it repeats blocks of convolutional layer, rectified linear units (ReLU) activation function, max-pooling layer, and up-sampling layer. The encoder extracts the feature and decreases the spatial dimensions of the input using the pooling operation, which can significantly reduce the computation of the network. The decoder, consisting of a series of up-sampling operations, aims to recover the dimensions. Some skip connections connect the corresponding feature maps from the encoder to the decoder, facilitating the propagation of detailed features.

#### 2.1.2. Deep Residual Network-18 (ResNet-18)

**Figure 1:**
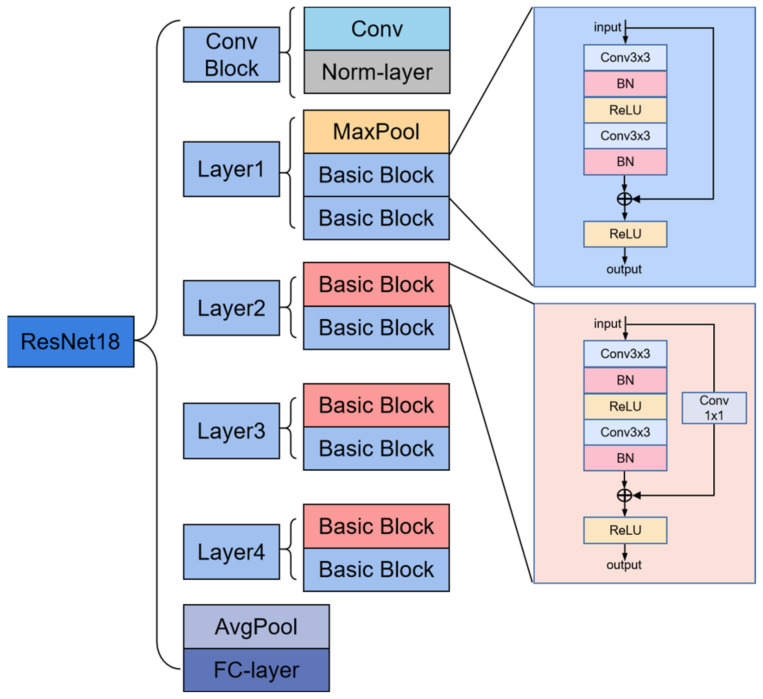
The structure of ResNet-18.

The ResNet-18, based on the CNN framework, is mainly composed of multiple residual blocks that enable the network to learn residual functions and facilitate the training of extremely deep networks [32]. The structure is shown in Figure 1, in which the first convolution block performs the convolution operation on the input for extracting low-level features. Then, the framework primarily consists of a stack of residual blocks that contains multiple convolutional layers with a skip connection. These blocks enable the network to maintain shorter paths and the gradients to bypass one or more layers. Specifically, the convolutional layer conv 1 × 1, as in Figure 1, is used in the basic block to adjust the input and output dimensions. The ReLU activation function introduces non-linearity and helps the network model complex relationships between features. There exist two pooling layers, max-pooling and avg-pooling, and the former reflects an essential clue about distinctive object features while discarding less significant information to reduce the spatial dimensions of the input. Instead, the latter considers all the pixel points in the given input map. The fully connected (FC) layer performs high-level feature extraction, which can combine the nonlinear features to gain the prediction.

#### 2.1.3. ShuffleNet-V2

The ShuffleNet-V1, an efficient neural network proposed by Zhang et al. [33], consists of channel shuffle, pointwise group convolution, and depthwise group convolution, where the channel shuffle operation is beneficial for information exchange between different groups of channels and the group convolution can decrease the computational cost. The ShuffleNet-V2 is improved based on the ShuffleNet-V1 by introducing a new operation, channel split, which splits the input into two branches in the channel dimension to decrease the memory access cost (MAC) [34]. Using the channel split operation, half of the feature channels input the next block directly, which can make full use of the feature. Instead of the “add” operation, the network leverages concatenate operations to connect two branches. Then, the result of the two branches is channel-shuffled to mix the features and ensure the two branches’ information is communicated. Compared with models such as ShuffleNet-V1 and DenseNet, ShuffleNet-V2 is faster and more accurate in processing.

#### 2.1.4. Temporal Convolutional Network (TCN)

The TCN network is improved based on the classical CNN by introducing causal convolution with a unidirectional structure [35]. At the same time, it applies the dilated convolution to increase the perceptual field so that the convolutional layer acquires a more extensive range of information. The TCN network replaces the convolutional layer with a residual convolution block containing two convolution layers and nonlinear mapping layers. Furthermore, a weight normalization, WeightNorm, and a spatial dropout, Dropout, are added in each convolutional layer to regularize the network.

**Figure 2:**
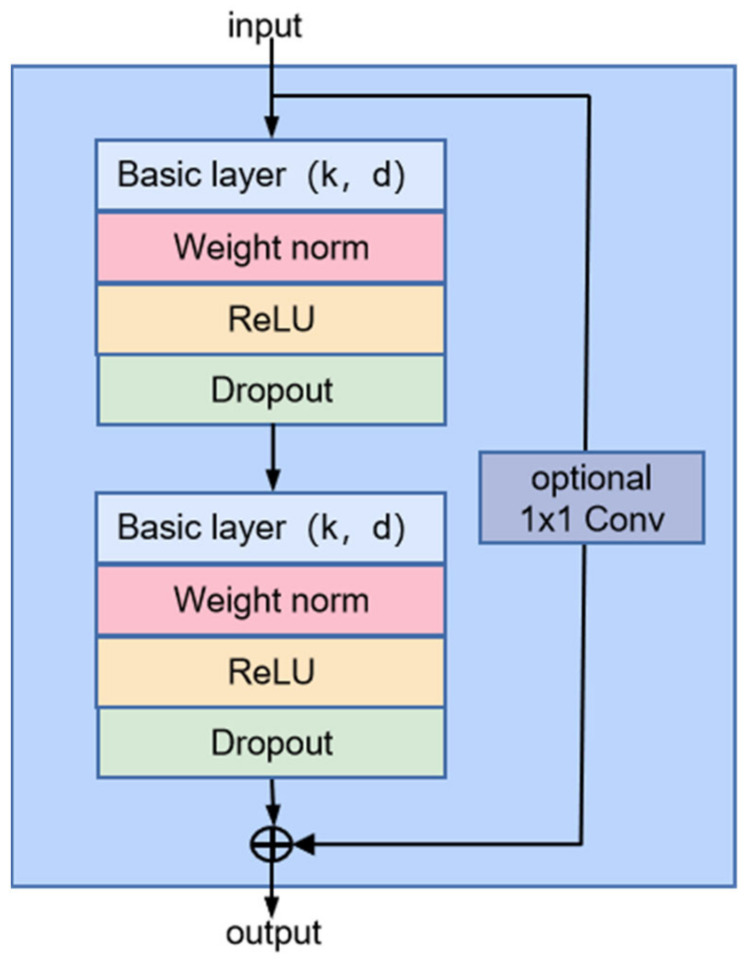
The structure of the residual convolutional block of TCN.

The specific structure is shown in Figure 2. The basic layer is a dilated casual convolutional layer, which is defined as the following formula.

(2) $$F\left(s\right)=\left(X{*}_{d}\;f\right)\left(s\right)=\sum_{i=0}^{k-1}f\left(i\right)\cdot X_{s-d\cdot i}$$

where, X is a one-dimension sequence input and f denotes the filter that applies to a region larger than itself by skipping part of the input. *d* accounts for the dilation factor (the larger dilation represents a broader range of receptive fields). k is the filter size, and s−d·i is the direction of the past. s is an element of the sequence input.

### 2.2. Training Target

In speech separation based on deep learning, choosing an effective training target is crucial for learning and generalization. Many models learn the complex functional relationship between mixed speech and clean speech to achieve separation. Previously, classical targets include mapping-based and masking-based. The former reflects a clean speech signal or its time-frequency representation, and the latter describes the relationship between the clean speech spectrogram and the noise speech spectrogram. It has been indicated that speech separation models using masking-based targets outperform those models that generally use mapping-based [36]. The ideal binary mask (IBM), a masking-based target, is used in supervised speech separation as a training target, which markedly improves audio intelligibility in noise [37]. The IBM is a binary mask, meaning it only estimates whether the signal exists or not, so it cannot recover the amplitude information. In contrast, the ideal ratio mask (IRM) can consider the magnitude of signals in the time-frequency domain. However, obtaining accurate information in practice is difficult, which complicates the implementation of IRM [38].

Instead of the IBM and IRM, which are commonly used in supervised speech separation tasks, Wang et al. proposed the complex ideal ratio mask (cIRM), which uses the magnitude and phase information of the speech signal to obtain a mask in the complex domain and tends to yield a better clean speech signal [39]. The cIRM calculation formula is as follows.

(3) $$cIRM=\frac{X_{r}S_{r}+X_{i}S_{i}}{X_{r}^{2}+X_{i}^{2}}+i\frac{X_{r}S_{i}-X_{i}S_{r}}{X_{r}^{2}+X_{i}^{2}}$$

where $X_{r}$ and $X_{i}$ denote the real and imaginary components of mixed speech, respectively. $S_{r}$ and $S_{i}$ are the real and imaginary components of clean speech. The predicted spectrograms for the separated speech signals are obtained depending on the complex mask and the mixed speech by the following formula.

(4) S=cIRM∗X

where S, X and ∗ denote the short-time Fourier transform (STFT) of separated speech, mixed speech, and complex multiplication, respectively.

## 3. Methods

**Figure 3:**
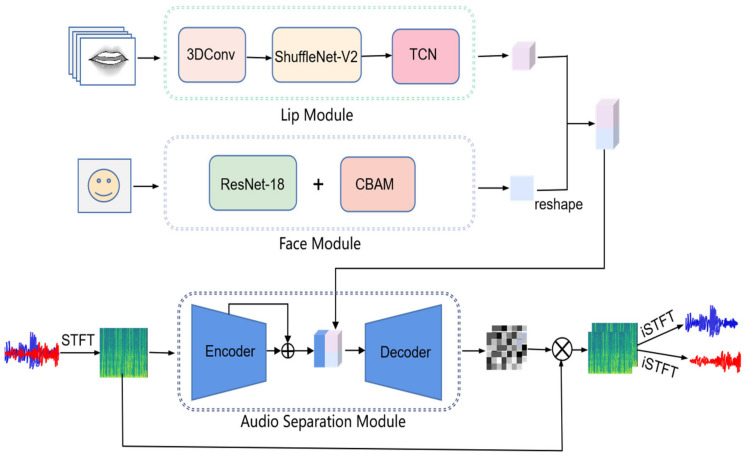
The architecture of our model.

The facial information and lip movement information of the speakers are extracted from the video to assist the speech separation model in estimating the mask of speakers A and B. The facial features are extracted from the video frame by the face module composed of ResNet-18 combined with the CBAM. The lip motion information is extracted from the lip action video stream by the lip module, which consists of 3D convolution, ShuffleNet-V2, and TCN. The facial features are reshaped and combined with the lip motion as the final visual feature input to the audio separation module. This module, which includes an encoder and a decoder, obtains the audio feature from the mixed audio and combines the visual to yield the target audio. The overall structure of the audio-visual speech separation model is shown in Figure 3.

### 3.1. Audio Separation Module

**Figure 4:**
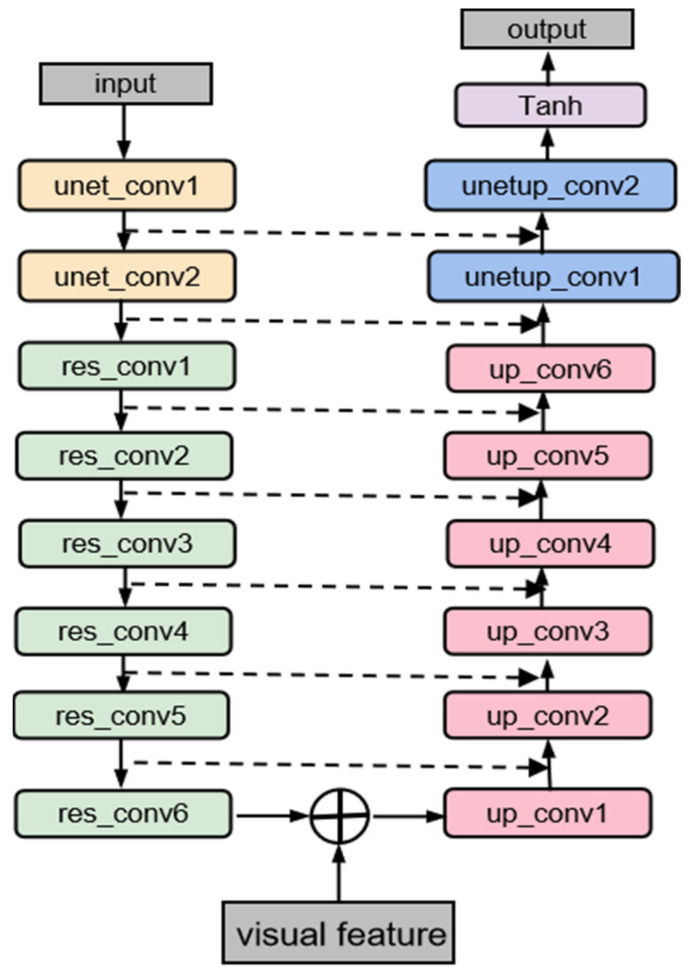
The structure of the improved U-Net. The encoder part mainly comprises two unet_conv blocks and six res_conv blocks. The decoder part mainly comprises two unetup_conv blocks with the same structure as unet_conv and six up_conv blocks.

The audio separation module is implemented based on a U-Net, as shown in Figure 4.

As one of the essential parts of the audio separation module, the encoder down-samples the mixed audio input first, which is achieved through two convolution blocks, unet_conv. This block consists of a convolutional layer, a batch normalization (BN) layer, and a ReLU activation function, and it can be defined as the following formula:

(5) $$Y=ReLU\left(BN\left(C^{4\times 4}\left(X\right)\right)\right)$$

where X and Y denote the input and output of the convolution block. $C^{4\times 4}$ is a convolution with a kernel size of 4 × 4. ReLU is a rectified linear units activation function, and BN is a batch normalization layer.

The two blocks downsample the dimension of frequency and time. After that, six convolution blocks, res_conv, are applied. However, different from the convolution block structure of the original U-Net, some modifications are introduced to improve the architecture. In each convolution block, a BN layer is applied between the convolutional layer and ReLU activation function to accelerate the network training, which has been proven effective [40,41]. The skip connection mechanism described in [32] is introduced to the convolution block in the encoder part, supporting the network to leverage better small audio feature details that are not easily distinguishable [30]. The effect of this mechanism is to express the network output as a linear sum of a nonlinear transformation of the input and the input, which the following equation can express.

(6) $$y=F\left(x\right)+x$$

where x and y denote the input and output of the layers, respectively. The function $F\left(\cdot \right)$ is the learned mapping of the residual connection.

The complex spectrogram of the mixed speech, X, inputs the encoder part to obtain the downsampled audio feature, $F_{a\_e}$:

(7) $$F_{a\_e}=Encoder\left(X\right)$$

After the residual convolution blocks, the downsampled audio feature, which has a dimension of 512, consolidates with the visual feature, $F_{v}$, in the temporal dimension to form the audio-visual feature, $F_{a\_v}$.

(8) $$F_{a\_v}=F_{a\_e}+F_{v}$$

The decoder has a similar structure to the encoder, except that the residual connections are not applied to the up-sampling convolution, up_conv, block. The up-sampling convolution block of the decoder part consists of an up-sampling layer, a convolutional layer, a BN layer, and a ReLU activation function, which can be defined as the following formula:

(9) $$Y=ReLU\left(BN\left(C^{3\times 3}\left(Upsample\left(X\right)\right)\right)\right)$$

where X and Y denote the input and output of the up-sampling convolution block. Upsample is the up-sampling layer, and $C^{3\times 3}$ is a convolution with a kernel size of 3 × 3.

The fusion audio-visual feature enters the decoder and then the output of the decoder, $F_{a\_d}$, is fed into the next Tanh layer. Then, the final prediction mask, M′, can be extracted.

(10) $$F_{a\_d}=Decoder\left(F_{a\_v}\right)$$

(11) $$M\prime =Tanh\left(F_{a\_d}\right)$$

where Tanh is the Tanh activation function used to adjust the mask value in the range of −1 to 1, and the prediction mask is multiplied by the spectrogram of the mixed speech. Then, the clean speech of the speaker can be recovered after the inverse STFT (iSTFT).

### 3.2. Visual Module

The visual feature consists of static facial information and continuous movement of the lip. They are extracted by the face module and lip module, respectively.

#### 3.2.1. Face Module

**Figure 5:**
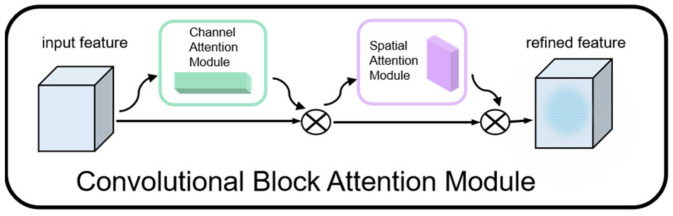
CBAM.

For facial information, the face module, based on the ResNet-18 network, takes a video frame as input. Considering that the video frame includes other parts besides the face, they are worthless for the separation process. As a result, the convolution block attention mechanism (CBAM) [42], as shown in Figure 5, is added to help this module to extract more critical face features. CBAM contains a channel attention module and a spatial attention module.

**Figure 6:**
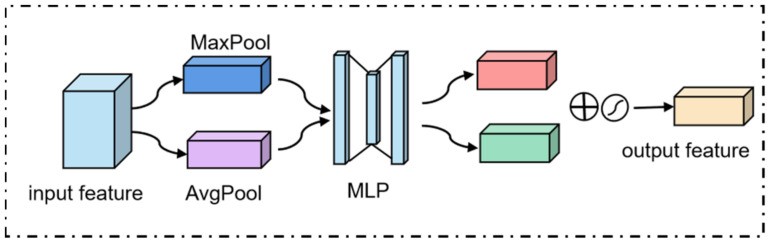
Channel attention module.

The channel attention module focuses on the visual input’s significant information and compresses it in the spatial dimension shown in Figure 6.

The avg-pooling layer considers all the pixel points in the given input map, and the max-pooling layer reflects an essential clue about distinctive object features. The output features are then fed into the shared multi-layer perceptron (MLP) to obtain the final channel attention features. In short, the process can be expressed by this equation:

(12) $$M_{c}\left(F\right)=\sigma \left(MLP\left(AvgPool\left(F\right)\right)+MLP\left(MaxPool\left(F\right)\right)\right)=\sigma \left(W_{1}\left(W_{0}\left(F_{avg}^{c}\right)\right)+W_{1}\left(W_{0}\left(F_{max}^{c}\right)\right)\right)$$

where MLP is the shared multi-layer perceptron, and F is the input of this module. $M_{c}\left(F\right)$ is the channel attention map. $W_{0}$ and $W_{1}$ are the weights of the MLP, which are shared for both inputs. $F_{avg}^{c}$ and $F_{\max}^{c}$ are the output features of the avg-pooling layer and max-pooling layer, respectively. σ is the sigmoid function.

**Figure 7:**
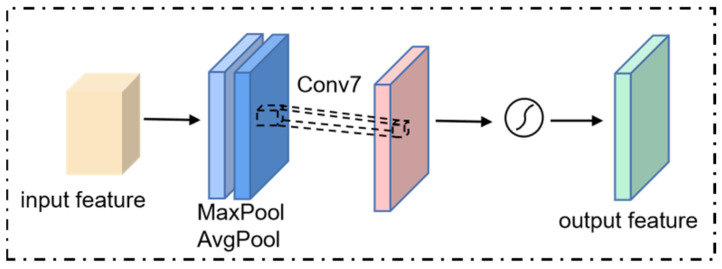
Spatial attention module.

The spatial attention module, complementary to the channel attention module, concentrates on the significant areas in the visual input and utilizes the inter-spatial relationship of features to gain a spatial attention map. The structure is shown in Figure 7.

The spatial attention mechanism performs global max-pooling and global ave-pooling operations on the input feature maps to extract the maximum and average values of features in the channel separately. After that, the output maps of the pooling layer are concatenated and then produce the final spatial attention feature by applying a convolutional layer and a sigmoid activation function. The process can be computed using the following formula:

(13) $$M_{s}\left(F\right)=\sigma \left(C^{7\times 7}\left(\left[AvgPool\left(F\right);MaxPool\left(F\right)\right]\right)\right)=\sigma \left(C^{7\times 7}\left(\left[F_{avg}^{s};F_{max}^{s}\right]\right)\right)$$

where $C^{7\times 7}$ is a convolutional layer with a kernel size of 7 × 7, and F is the input of this module. $M_{s}\left(F\right)$ is the channel attention map. $F_{avg}^{s}$ and $F_{max}^{s}$ are the output features of the avg-pooling layer and max-pooling layer, respectively. σ is the sigmoid function.

**Figure 8:**
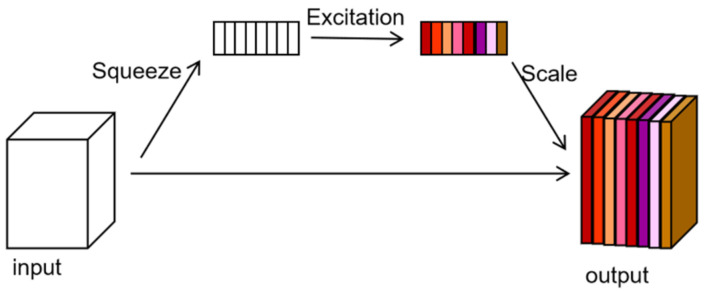
The structure of the SE attention mechanism.

Apart from the CBAM, the squeeze and excitation (SE) attention mechanism [43] is widely used in semantic segmentation and object direction fields. It mainly consists of two operations, squeeze and excitation, intending to explicitly model the relationship between the feature channels. Firstly, the squeeze operation performs on the input feature map to encode the spatial features of a channel as a global feature, which is achieved by global average pooling. Then, the excitation operation is applied to the global feature to learn the relationship between channels and get the weights of different channels. Finally, the scale operation is performed so that the obtained weights of different channels are multiplied by the original feature map to obtain the final features. The SE attention mechanism is shown in Figure 8.

**Figure 9:**
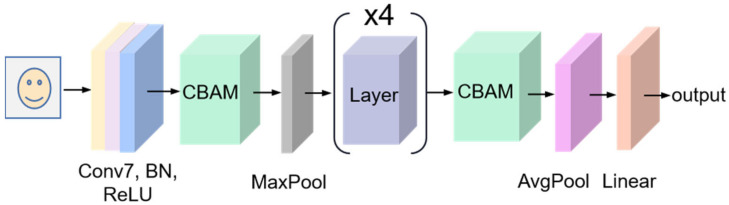
The structure of the face module.

The face module incorporating the attention mechanism could focus more on the differences and similarities between the visual features of different speakers. The specific structure of the face module is shown in Figure 9. The first block of this module is a convolution block consisting of a convolution with a kernel size of 7 × 7, a BN layer, and a ReLU activation function. The other components are two CBAM layers, a max-pooling layer, four residual layers, an avg-pooling layer, and a linear layer. Since the basic block of the network is desired not to be changed, the first CBAM layer is added after the ReLU layer, and the second one is applied before the avg-pooling layer.

Let $f_{face}$ and $F_{face}$ represent the input feature of the face module and the input feature of the first attention mechanism layer of the face module, respectively. $F_{face}$ is obtained by the following calculation.

(14) $$F_{\mathrm{face}}=ReLU\left(BN\left(C^{7\times 7}\left(f_{\mathrm{face}}\right)\right)\right)$$

where the $C^{7\times 7}$ is a convolution with kernel size of 7 × 7.

#### 3.2.2. Lip Module

For lip information, the SFD face detector is applied to capture the crucial points of the face. The mouth area being grayscale processed inputs to the lip module, which firstly passes through a 3D convolution block consisting of 3D convolution, BN, ReLU, and 3D max-pooling layers. Then, the output feature of the 3D convolutional layer inputs ShuffletNet-V2 and a temporal convolutional layer (TCN) sequentially, like [44,45], to extract the final lip motion features.

Let $f_{lip}$ represent the input feature of the lip module and $F_{lip}$ as the input feature of the ShuffleNet-V2 network. Then, the $F_{lip}$ can be converted from three dimensions to two dimensions and described as follows.

(15) $$F_{\mathrm{lip}}={\mathrm{Max}}^{5\times 7\times 7}\left(ReLU\left(BN\left(C^{5\times 7\times 7}\left(f_{\mathrm{lip}}\right)\right)\right)\right)$$

where the $C^{5\times 7\times 7}$ is the 3D convolutional layer with a kernel size of 5 × 7 × 7 and $Max^{5\times 7\times 7}$ denotes the 3DMaxpool operation.

$F_{face}^{\prime }$ and $F_{lip}^{\prime }$ denote the output of the face module and lip module, respectively. $F_{v}$ is the final visual feature that fuses the face and lip features. The fused visual feature is obtained as the following formula.

(16) $$F_{v}=\mathrm{concat}\left(F_{face}^{\prime },\;F_{lip}^{\prime }\right)$$

### 3.3. Training Loss

To perform the separation, the proposed model predicts the cIRM of the audio to separate clean speech corresponding to the speaker from the mixed audio signal. The audio separation module optimizes the network by computing the Huber loss of the true and the predicted mask. Huber loss avoids the disadvantage of L1 loss being computationally intensive and L2 loss being difficult to derive zero points [46]. The formula is as follows.

(17) $$L_{A}=\left\{\begin{array}{c} \frac{1}{2}{\left(M^{\prime }-M\right)}^{2},\;\left|M^{\prime }-M\right|\leq \delta \\ \delta \left|M^{\prime }-M\right|-\frac{1}{2}\delta^{2},\;otherwise \end{array}\right.$$

where the $M^{'}$ is the true mask, and the M is the predicted one. The δ is a parameter of Huber loss, which uses squared error when the prediction deviation is less than δ, and linear error when the prediction deviation is greater than δ.

**Figure 10:**
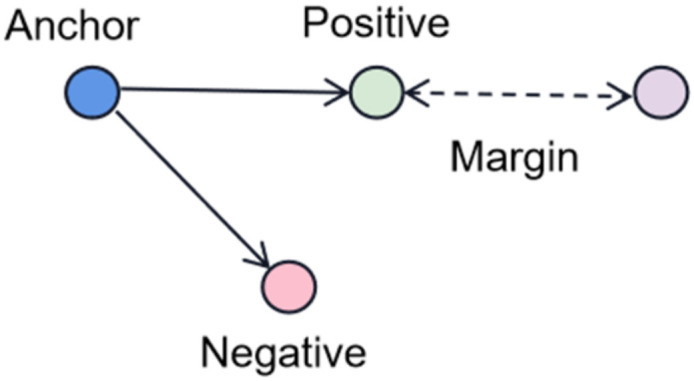
Triplet loss.

It has been established that speaker similarity and separation performance are inversely proportional [25]. Therefore, how to leverage visual information related to audio information to boost performance is an essential issue. The similarity of speaker visual and audio embeddings is considered in this model and calculated using Triplet loss [47]. The diagram of Triplet loss is shown in Figure 10.

Where the positive is a sample in the same category as the anchor. Negative is a sample in a different category from the anchor, and the margin is a non-zero constant. Triplet loss calculates the similarity between samples by optimizing the distance between the anchor and positive to be less than the distance between the anchor and negative. In this paper, the visual embeddings $f_{A}$ and $f_{B}$ are the ResNet-18 network outputs of the face image frames and the audio embeddings $a_{A}$ and $a_{B}$ are the ResNet-18 network outputs of the clean speaker speeches. The final optimization purpose is to make the distance between the audio and face of the same speaker close and the different one far. A margin is used to distinguish their facial features when they are at the same distance. The cross-modal audio-visual loss, $L_{AV}$, is calculated as follows:

(18) $$L_{AV}=\max\left\{D\left(a_{A},f_{A}\right)-D\left(a_{A},f_{B}\right)+m,0\right\}+\max\left\{D\left(a_{B},f_{B}\right)-D\left(a_{B},f_{A}\right)+m,0\right\}$$

The whole training function of this model combines the above two losses, which are illustrated as follows.

(19) $$L=\lambda_{1}L_{A}+\lambda_{2}L_{AV}$$

where $\lambda_{1}$ and $\lambda_{2}$ are the weight for the cIRM mask loss and audio-visual loss, respectively.

## 4. Experiments

### 4.1. Dataset

The proposed model is trained on the VoxCeleb2 dataset, which contains over 1 million audios for over 6000 celebrities and corresponding videos [48]. The speakers are from a wide range of different nationalities. The gender distribution of the dataset is relatively even, with 39% of the speakers being women. The dataset additionally offers facial detections and tracks of the speakers.

The videos in the dataset are obtained in plenty of natural environments, including interviews on the red carpet, in outdoor stadiums, and studios. Correspondingly, some audio in these videos is unclear, which contains some background segments like chatter, laughter, and varying room acoustics, making the audio closer to the actual scenario. The length of the audio varies from 4 s to 20 s, where most are shorter than 10 s. In the experiment, the videos of a speaker are randomly selected as the video data. For audio data, FFmpeg is used to obtain each speaker’s audio from video and save it to a WAV format.

### 4.2. Setup

The framework is Pytorch 1.11.0 with CUDA version 11.1. The mixed speech is composed of single audio that randomly summed two speakers’ speech signals and sampled them at 16 kHz, a common standard for audio sampling that ensures the signal is perfectly reconstructed. Then, a complex spectrogram of the mixed speech, by computing the STFT of speech, inputs to the audio separation module. The dimensions are 2 × F × T, where F and T are the frequency and time dimensions of the spectrogram, and in this paper, the size of each spectrogram is 2 × 257 × 256. For the relevant parameter settings of STFT, Hann window is used for computing STFT with 400 window length, 160 hop size, and 512 FFT window size. The audio is a 2.55 s clip with a 16 kHz sampling rate. The image size of the input face module is 224 × 224, which is downsampled to a 128-dimensional facial feature after the ResNet-18 network. Then, the facial feature is reshaped along the time dimension to concatenate with the lip feature. A shot face detector (SFD) is used to detect facial landmarks from the face track. Then, an 88 × 88 region of interest is cropped when the center of the mouth is located. The input to the lip module is 64 frame grayscale images, and each frame size is 88 × 88. The lip module extracts a feature of lip motion of dimension 512 × 64, which concatenates with the reshaped facial feature to gain a final visual feature of dimension 640 × 64.

We empirically select parameters by tuning on initial performance. The Adam optimizer is used for optimizing the model, and the weight decay is set to 0.0001. The learning rates of the face, lip, and audio separation modules are set to 0.00001, 0.0001, and 0.0001, respectively. Speech and visual information have different effects on the separation results. The parameters $\lambda_{1}$ and $\lambda_{2}$ in Formula (19) are set to 0.1 and 0.01. The hyperparameter δ in Formula (17) is set to 0.5, and the parameter m in Formula (18) is set to 0.5 as well.

### 4.3. Results

In speech separation tasks, SDR, PESQ, and STOI are commonly used as a prominent set of evaluation metrics. SDR represents the source-to-distortion ratio. Short-time objective intelligibility (STOI) measures the correlation between the short-term time envelope of the reference speech and the separated speech [49]. For speech quality, perceptual evaluation of speech quality (PESQ) is the standard metric [50], which applies an auditory transformation to produce a loudness spectrum and compares the loudness spectrum of the clean reference signal with that of the separated signal. The key point to emphasize is that SDR and PESQ are influenced by audio characteristics such as volume. Additionally, PESQ is an objective assessment standard that approximates subjective experiences, but it may deviate from human subjective perceptions in certain scenarios. Concerning STOI, the measure of speech intelligibility in the real world is related to human attention levels, implying the need to account for the energy expended in processing the sound. However, quantifying individual energy expenditure is challenging, rendering this metric impractical in reality. Taken together, one evaluation metric cannot fully represent human perception. Therefore, this paper evaluates the separation quality of the model more objectively by considering multiple metrics. This paper also uses source-to-interference ratio (SIR) and source-to-artifacts ratio (SAR) as evaluation metrics for the proposed model. The SDR score is obtained by calculating the speech mixture and the estimated clean speech. The two-speaker mixtures are analyzed, and the results by different configurations are compared.

CBAM is introduced to the face module to evaluate the attention mechanism’s effect. In addition, we also compare the effect of the squeeze-and-excitation (SE) attention mechanism. According to Table 1, adding CBAM layers is better than no attention, which illustrates that the CBAM helps the face module extract facial information accurately and further validates the idea that the face contains crucial features for the separation task. However, compared with the CBAM, the addition of SE does not enhance the separation performance, which may be because the CBAM has an additional max-pooling layer for parallel computation, which makes it lose less information and acquire more information by connecting max-pooling and avg-pooling in parallel than using the avg-pooling layer alone, so the CBAM is more effective than SE.

Table 2 shows the results of the U-Net with different residual connections. When degradation occurs in the deep network, the residual connection is a bridge to pass information from one layer to the next. The results also indicate that the improved network outperforms the U-Net without the contribution of residual connections. According to Table 2, the separation performance is significantly advanced with the number of residual connections increasing. Specifically, the six-residual model is about 1.2 dB higher than no residual one, and it can be seen that the scores of PESQ and STOI are higher than no residual one at the same time.

This paper compared the separation performance of the present model with the previous audio-visual separation methods. As Table 3 exhibits, the proposed model outperforms the conventional method in terms of metrics like PESQ, STOI, and SDR, which indicates that the proposed model improves the quality of separation to some extent. The scores of SIR and SAR are also higher than the lip-and-face model [27]. Our method advanced the performance by adding residual connections to the encoder for detailed information and extracting more essential face features by attention mechanism. The STOI and SDR of the proposed model are better than the lip-only methods [26,29], which may result from different visual features. Consequently, the effectiveness of the model separation varies slightly when different visual cues are introduced. The dual visual cues used in this paper allow for more comprehensive use of speaker visual and speech information and achieve better separation performance than using mouth movements or face alone.

**Figure 11:**
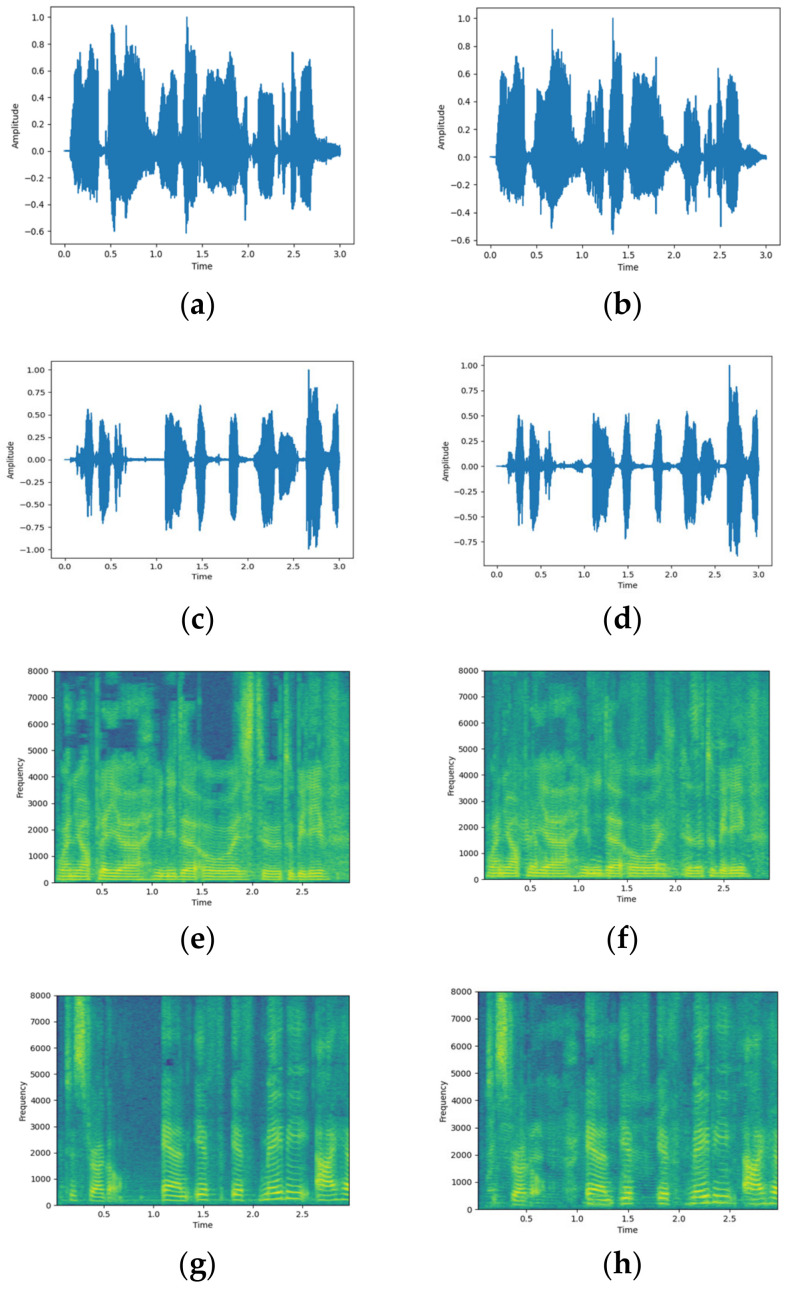
The waveform and spectrogram graph of the audio. (**a**,**c**) are the waveforms of the source audio signal; (**b**,**d**) are the waveforms of the separated audio signal; (**e**,**g**) are the spectrograms of the source audio signal; (**f**,**h**) are the spectrograms of the separated audio signals.

The qualitative illustration of the proposed separation model is shown in Figure 11. By comparing the separated waveforms and spectrograms with the source audio signal, it can be observed that our model successfully separates the clean speech signal from the mixture, and the separated results are similar to the clean speech signal, showing the effectiveness of the method.

## 5. Conclusions

This paper proposes an audio-visual speech separation model with dual visual cues, which contains lip, face, and audio separation modules. The residual connection introduced in the audio separation module strengthens the extraction of detailed features because it can pass information in layers to advance the learning feature ability and solve gradient degradation in deep networks. For the face module, a CBAM attention mechanism helps it focus on the crucial facial features. The result denotes that the CBAM is beneficial for feature extraction and improves the separation performance. The training objective additionally considers the similarity between facial and audio features, enabling the model to exploit the complementary cues of vision and speech, and the fusion of mouth dynamic movements and facial static feature enables the visual information to be fully leveraged. Although the experiments show that the model achieves positive performance, it is undeniable that the method still has some shortcomings compared with humans. The process of human auditory perception may involve the processing of other visual information, such as facial expressions, gaze, and head movements. Facial expressions, in particular, serve as a universal language that can reflect the emotions and attitudes of the speaker. In future work, considering this information may prove advantageous for speech separation. Furthermore, spatial information, such as the direction and distance of sound sources, may also affect the performance of speech separation algorithms. Therefore, we intend to consider the other cues to promote the further application of speech separation.

## Figures and Tables

**Table 1:** The results of SDR, PESQ, and STOI with different attention mechanisms added. It should be noted that the results shown in this table are implemented on the U-Net without residual connection.

Methods	SDR	PESQ	STOI
No attention	8.47	2.51	0.78
Add SE	8.45	2.52	0.78
Add CBAM	8.96	2.53	0.82

**Table 2:** The results of SDR, PESQ, and STOI with different numbers of residual connections added.

Methods	SDR	PESQ	STOI
No-residual	8.96	2.53	0.82
Two-residual	9.41	2.56	0.82
Six-residual	10.1	2.60	0.85

**Table 3:** Comparison with existing AV speech separation methods on the VoxCeleb2 dataset. The author’s results in their paper are directly used for comparisons.

Methods	SDR	PESQ	STOI	SAR	SIR
Ito et al. [26]	6.16	2.44	0.74	-	-
Li and Qian [29]	6.7	2.57	-	-	-
Deng and Wei [27]	7.58	2.55	0.81	12.12	10.13
Only face	7.15	2.46	0.76	11.02	10.36
Only lip	9.84	2.58	0.82	11.71	14.82
This model	10.1	2.60	0.85	12.20	16.62

## Data Availability

Not applicable.
